# Electrode Impact on the Electrical Breakdown of Dielectric Elastomer Thin Films

**DOI:** 10.3390/polym15204071

**Published:** 2023-10-12

**Authors:** Bettina Fasolt, Fabio Beco Albuquerque, Jonas Hubertus, Günter Schultes, Herbert Shea, Stefan Seelecke

**Affiliations:** 1 Intelligent Material Systems Lab, Center for Mechatronics and Automation Technology, ZeMA gGmbH, DE-66121 Saarbrücken, Germany; 2 Intelligent Material Systems Lab, Department of Systems Engineering, Department of Materials Science and Engineering, Saarland University, DE-66121 Saarbrücken, Germany; stefan.seelecke@imsl.uni-saarland.de; 3 LMTS Soft Transducers Laboratory, EPFL Ecole Polytechnique Fédérale de Lausanne, CH-2002 Neuchâtel, Switzerlandherbert.shea@epfl.ch (H.S.); 4 Sensors and Thin Film Group, University of Applied Sciences, DE-66117 Saarbrücken, Germany; jonas.hubertus@htwsaar.de (J.H.); guenter.schultes@htwsaar.de (G.S.)

**Keywords:** dielectric breakdown test, electrode manufacturing methods, influence electrodes, silicone films, carbon black, environmental conditions

## Abstract

Dielectric Elastomer Actuators (DEAs) enable the realization of energy-efficient and compact actuator systems. DEAs operate at the kilovolt range with typically microampere-level currents and hence minimize thermal losses in comparison to low voltage/high current actuators such as shape memory alloys or solenoids. The main limiting factor for reaching high energy density in high voltage applications is dielectric breakdown. In previous investigations on silicone-based thin films, we reported that not only do environmental conditions and film parameters such as pre-stretch play an important role but that electrode composition also has a significant impact on the breakdown behavior. In this paper, we present a comprehensive study of electrical breakdown on thin silicone films coated with electrodes manufactured by five different methods: screen printing, inkjet printing, pad printing, gold sputtering, and nickel sputtering. For each method, breakdown was studied under environmental conditions ranging from 1 °C to 80 °C and 10% to 90% relative humidity. The effect of different manufacturing methods was analyzed as was the influence of parameters such as solvents, silicone content, and the particle processing method. The breakdown field increases with increasing temperature and decreases with increasing humidity for all electrode types. The stiffer metal electrodes have a higher breakdown field than the carbon-based electrodes, for which particle size also plays a large role.

## 1. Introduction

The utilization of DEAs as electromechanical transducers is attractive for a number of different applications such as valves [1,2], pumps [3], and switches [4,5], as well as in haptic devices [6,7], wearables [8,9], and soft robotics applications [10,11,12,13,14]. DEAs are lightweight and offer silent, energy-efficient actuation without the use of rare earth materials. Additionally, the actuators’ self-sensing properties enable smart applications without the need for external sensors.

A standard DEA typically consists of a compliant dielectric elastomer membrane sandwiched between two stretchable electrodes [15,16]. When a voltage is applied to the electrodes, electrostatic forces lead to a reduction of the membrane thickness along with a simultaneous lateral expansion, thus resulting in voltage-controlled motion.

An important limiting factor for high voltage applications is dielectric breakdown. The breakdown field strength *E_BD* represents the maximum value of the electrical field which can be withstood by the membrane. The Maxwell pressure scales as *E_BD*^2^ while the elastic energy density scales as *E_BD*^4^ [17] It is thus essential to be able to operate DEAs at high electric fields and to understand on which parameters the maximal breakdown field depends. Environmental conditions such as temperature and humidity, as well as the pre-stretch of the membrane, and the dielectric material itself are only a selection of possible parameters known to have an influence on the breakdown voltage [18,19,20,21,22,23,24]. In a previous breakdown study by Fasolt et al. conducted on pure silicone film and film with screen-printed carbon black (CB) electrodes, it was discovered that in addition to the above parameters the electrode also had a significant impact on the breakdown, lowering the breakdown field by up to 20% [25]. To validate the influence of electrodes on the breakdown behavior, the results were compared with a study conducted by Albuquerque and Shea [16], which tested silicone film with sputtered gold electrodes under various environmental conditions. The results of the breakdown fields varied significantly but because the film thickness, pre-stretch, and electrode material were also different, a direct comparison was not possible. Other published studies about breakdown behavior of silicone thin film used yet different electrode materials: Förster-Zügel et al. used graphite powder and a shadow mask [26], Stoyanov et al. sprayed carbon nanotubes [27], Jiang et al. applied conductive carbon grease [28], Albuquerque and Shea applied CB electrodes by pad printing [29], and Zakaria et al. sputtered silver electrodes [30]. In order to establish a framework for transferability of measured results between different studies, the current paper systematically investigates the effect of different electrode materials and electrode manufacturing methods over a wide temperature and humidity range.

Specifically, this study provides a comprehensive breakdown investigation conducted on electrodes applied by four different manufacturing methods, using the same test setup and environmental conditions, ranging from 1 °C to 80 °C and 10% to 90% relative humidity. Three research groups collaborated on this project and provided different electrodes manufactured with application methods used in their labs: screen printing, inkjet printing, pad printing, gold sputtering, and nickel sputtering. The electrodes were applied on the same 20 µm-thick silicone dielectric material, Wacker Elastosil 2030/20 µm, and the same bi-axial pre-stretch of the film was used for all samples.

Each manufacturing method has its unique application scope, shown in Table 1. Manufacturing methods such as spraying, spin coating, blade casting, or 3D printing are also possible but were not included in this study [31,32,33]. Sputtered metal electrodes have a high conductivity and nanometer-scale thicknesses but usually lose conductivity when stretched. Hubertus et al. [34,35,36] describe a method where electrodes are sputtered on a pre-stretched film and subsequently released so that they exhibit a strongly wrinkled configuration, enabling subsequent stretching within the pre-stretch range and even above. Carbon black electrodes are attractive low-cost materials and can be applied by high-throughput and scalable processes such as screen printing, pad printing, or inkjet printing. These electrodes can remain conductive even at large deformations and hence are a widely used material for soft actuator and sensor applications. Other printable electrode materials such as carbon nanotubes, silver, and graphenes are not included in the study.

The breakdown behavior of the different manufacturing methods is systematically analyzed in this paper and possible breakdown-affecting parameters are discussed. As a reference, experiments were also conducted on samples without applied electrodes. The investigations conducted in this paper are divided into three main sections. The focus of the first group is the influence of environmental conditions such as temperature and humidity on the breakdown behavior. The results are shown for all different types of electrodes. Section 3.1, Section 3.2 and Section 3.3 focus on the differences in breakdown behavior for the different types of electrodes. Influencing parameters such as the mechanical effect of the manufacturing method and stiffness (pull-in effect) are discussed. Section 3.4, Section 3.5 and Section 3.6 examine possible influencing parameters for the carbon black-based electrodes such as solvents, carbon black processing (mixing and milling), and silicone content. The experiments of Section 3.3, Section 3.4, Section 3.5 and Section 3.6 were conducted under standard environmental conditions (20 °C/55% rel. humidity).

## 2. Experimental Setup and Procedure

### 2.1. Test Setup

**Figure 1:**
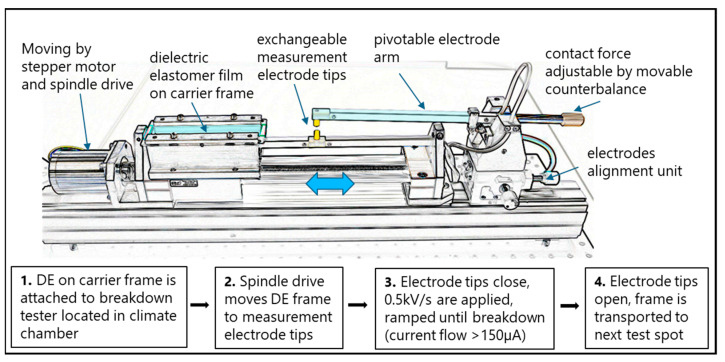
Schematic sketch (top) and diagram (bottom) of the breakdown tester and measurement steps.

The breakdown tests were conducted using a custom-built automated electrical breakdown test setup. A detailed description of the development and design is given in [37]. During the tests, two gold-plated electrodes, subsequently denoted measurement electrodes, with a diameter of 6.3 mm, one moveable on a pivoting arm and one fixed, make contact with the silicone film and voltage is applied at a rate of 0.5 kV/s until breakdown. The breakdown voltage is defined as the voltage when the current flow through the material reached a value of 150 µA. A LabVIEW test software automatically stops the voltage application and records the breakdown value when the admissible current flow is detected by the HypotMAX 7710 Dielectric Withstand Tester (Associated Research, Lake Forest, IL, USA). The movable top electrode is flat, and the bottom electrode has a convex shape with a radius of curvature of 26 mm. The geometry configuration flat top and flat bottom was also investigated for the same film and pre-stretch, but only a negligible difference in the breakdown field was detected. Therefore, the flat/convex shape, also compatible with standardization suggestions from Carpi et al. [38], was chosen for the measurement electrodes, as it also features minimal membrane interactions during spot positioning. After breakdown, the electrodes separate, and the tester automatically moves to the next position. The test setup was placed in a climate chamber Vötsch CLIMEEVENT C/600/40/3 to be able to conduct all breakdown tests in a controlled environment. The tester design allows for consecutive testing without the need to open the climate chamber. Figure 1 shows a schematic illustration of the breakdown tester and the steps carried out for each measurement point.

**Figure 2:**
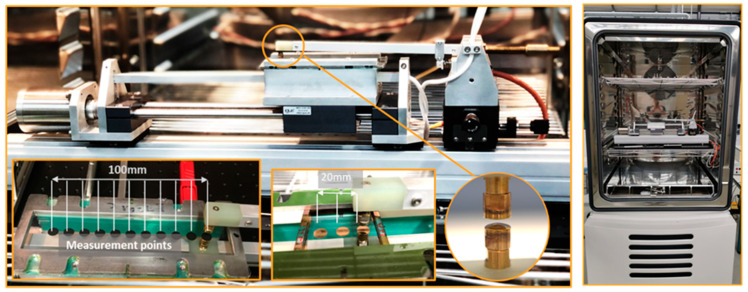
Left: Automated breakdown tester with measurement electrodes (convex/flat design for voltage application) and example of screen-printed and gold-sputtered electrodes. Right: Test setup in climate chamber CLIMEEVENT C/600/40/3, Fa. Vötsch.

The tests were conducted on pure film and film with applied electrodes. To compare these results, all manufactured electrodes had the same diameter as the measurement electrodes. Two different test frames were used. The frames for the screen-printed, nickel-sputtered, and pure film were prepared at the iMSL lab on a metal frame with eleven measurement points, and the gold-sputtered, pad-printed, and inkjet-printed electrodes were prepared at the EPFL lab on plastic frames with three measurement points each. Figure 2 shows a picture of the automated tester with the measurement electrodes and an example of the screen-printed and gold-sputtered electrode samples located in the tester as well as the placement of the tester in the climate chamber.

### 2.2. Materials and Sample Preparation

Wacker Elastosil 2030/20 µm, pre-stretched bi-axially by ʎ1 = ʎ2 = 1.3, is used as a dielectric film for all samples. The average sample thickness after stretching is 11.8 µm. The samples without electrodes, with screen-printed electrodes, and with nickel-sputtered electrodes are pre-stretched using an automated stretcher, consisting of two separately controlled stepper motors and vacuum clamps for the fixation of the DE film. Screen-printed samples are first transferred onto special printer frames to allow for the printing of two designs at the same time and are subsequently transferred onto the test frames. The nickel-sputtered samples and the samples used for tests without electrodes are directly transferred to the test frames used in the breakdown tester. This procedure is described in more detail in [37]. The gold-sputtered, inkjet-printed, and pad-printed samples are pre-stretched using a circular stretcher and transferred to PMMA frames coated with a pressure-sensitive adhesive (Adhesives Research ArClear). The thickness is controlled using a white light transmission interferometer, as described in [39].

#### 2.2.1. Gold-Sputtered Samples

The gold electrodes (≈20 nm thick) are applied over a mask using a Jeol JFC-1200 gold coater, Jeol USA, Inc., Peabody, MA, USA (Argon, 8 Pa, 140 s at 20 mA), as described in [40]. The coating is carried out on both sides, leading to circular 6.3 mm diameter electrodes.

#### 2.2.2. Nickel-Sputtered Samples

Ten nm-thick nickel electrodes are deposited by a DC magnetron sputter process in a laboratory vacuum chamber. At the beginning of the process, the samples, covered with a shadow mask, are inserted into the vacuum chamber and placed on a movable sample holder. The pumping process is started until a background pressure of less than 1 × 10^−5^ mbar is reached. Prior to sputtering, three pump–purge cycles are executed, where Argon is let into the chamber up to a pressure of 1 × 10^−1^ mbar and pumped out again. A constant gas flow of 15 sccm Argon in combination with an appropriate position of a downstream throttle then leads to a constant sputter pressure of 1.5 × 10^−3^ mbar. The sputtering process is started by first pre-sputtering the magnetron target for 1.5 min, while the sample is still located outside the influence of the target. After that, the sample is transferred under the target and coated with a 10 nm-thick nickel thin film. A total of 300 W is applied to the target and held constant during the whole process. With a target-to-substrate distance of 4.5 cm, a 10 nm-thick nickel thin film is manufactured in 5 s. The geometry of the deposited thin film is realized by using a shadow mask, lasering 11 circles with a diameter of 6.3 mm and a distance of 10 mm between the centers of the circles out of a 50 µm-thick metal foil.

#### 2.2.3. Screen-Printed Samples

A screen-printing process requires electrode materials for printing and a screen provided with the desired sample design [41]. The electrode material is prepared using a mixture of 83 wt.% solvent (50% Coats screen VD60, 50% Wacker Belsil DM 1 Plus), 3.8 wt.% carbon black, and 13.2 wt.% PDMS. The solvent is added to achieve the viscosity necessary for screen printing. The preparation of the electrode material is carried out in multiple steps. First, the carbon black and solvents are blended in a planetary mixer. This mixture is subsequently ground in a three-roll mill and, after adding PDMS, again blended in the planetary mixer. A screen with two sets of eleven dots, each with a diameter of 6.3 mm, is prepared, and the electrode material is screen printed onto the pre-stretched film, achieving a thickness of 3 µm. After heat curing at 150 °C for 10 min, the electrodes are screen printed onto the other side of the film and heat cured for another 60 min at 150 °C. The film is then transferred onto two test frames consisting of 11 electrode dots designed for testing in a breakdown tester. After curing, the solvents are evaporated, and the mixture consists of 25 wt.% carbon black and 75 wt.% silicone.

#### 2.2.4. Pad-Printed Samples

The carbon black-PDMS composite electrode is a 4 ± 1 µm-thick pad-printed electrode comprising 0.8 g of carbon black (Ketjenblack EC-300J, Nouryon, Amsterdam, Netherlands) dispersed in 8 g of silicone elastomer (Silbione LSR 4305, Elkem, Oslo, Norway) of A:B ratio 1:1 and 32 g of a mixture of 50% isopropanol and 50% iso-octan, prepared following Rosset et al. [39] using a planetary mixer. After pad printing, the samples with the pad-printed electrodes are cured at 80 °C for 1 h. The pad-printing process is repeated on the opposite membrane side, leading to 6.3 mm diameter electrodes.

#### 2.2.5. Inkjet-Printed Samples

The inkjet-printed electrodes (3 ± 1 µm thick) contain carbon black (Ketjenblack EC-300J), a dispersant (Wacker Belsil SPG 128 VP, WACKER Chemie AG, Munic, Germany), and a solvent (DOWSIL OS2, Dow Chemical Company, Midland, MI, USA) and were applied following the process described in Schlatter et al. [42]. Circular electrodes of 6.3 mm were directly printed on the membrane.

### 2.3. Test Procedure

For characterization under well-defined environmental conditions, the breakdown tester is placed in a climate chamber. The samples are tested at six different environmental conditions, covering a wide range of possible conditions from low via medium to high temperatures with low, medium, and high humidities, Table 2. The difference in water content between low and high humidity at 1 °C is only 4 g/m³, which is outside of the adjustable range of the climate chamber.

The respective conditions are adjusted, and when stable, an additional 30 min remain before the test samples are placed into the chamber. One test sample is immediately fixed onto the movable station of the breakdown tester and five more samples are placed on a storage rack located above the tester. After 60 min, thermal equilibrium is reached in the test samples, and the tests are conducted on all test points consecutively (eleven electrodes on the large frames and three on the small frames). The tested frame is removed and replaced with a sample from the storage rack, which will then be re-stocked with samples from outside the climate chamber so that the minimum dwell time for each sample is 1 h. When conditions are stable again after replacement, another 10 min remain before the next test is conducted. This procedure is repeated until results for 15 measurement points per condition are available.

## 3. Results

The motivation of this study was to understand how different electrode materials and their manufacturing methods influence dielectric breakdown behavior in thin silicone films. First, the influence of six different environmental conditions is discussed in Section 3.1. Then, Section 3.2 and Section 3.3 discuss the mechanical effects associated with the different electrode materials. This includes the mechanical impact due to the manufacturing method as well as the different stiffnesses of the metal and non-metal electrodes. These experiments are conducted at defined standard condition (20 °C/55% RH). In Section 3.4, Section 3.5 and Section 3.6, the focus is on the carbon black-based electrodes, which are of particular relevance as they are not only used for lab breakdown measurements but also in applications with dielectric elastomer actuator devices. Here, parameters important for manufacturing, i.e., solvents, carbon black processing, and silicone content, and their impact on the breakdown behavior are systematically studied.

**Figure 3:**
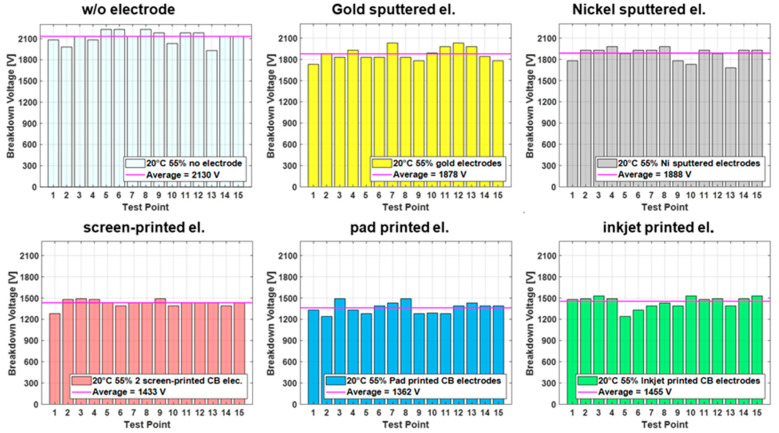
Breakdown voltage of Elastosil 2030/20 at 20 °C 55% RH for all electrode types, with 15 measurement points per sample.

Results from the breakdown measurements performed for each test case typically lead to plots such as the one shown in Figure 3, displaying breakdown results for all electrode types at standard conditions. Each test point is consecutively tested by connecting the setup electrodes of the breakdown tester to the manufactured top and bottom electrode and applying a voltage. The voltage is ramped with 500 V/s until breakdown, which is defined by a current flow of >150 µA through the dielectric material. The breakdown voltage is recorded for each breakdown point in the tested sequence until 15 points are tested. The results in Figure 3 indicate significant differences between the breakdown voltages for metal-sputtered electrodes and carbon black (CB) electrodes, showing an average of more than 400 volts (>20%) higher for the sputtered electrodes than the CB electrodes. The samples without electrodes are tested as a reference and are consistently higher than for all the samples with applied electrodes.

However, to compare the different conditions and electrodes, a bar graph illustration is impractical. Therefore, for the remaining figures in this paper, a boxplot design is used, which allows for a compact comparison and interpretation of the different cases. For example, the breakdown of single spots (depicted as red cross) due to imperfections in the film are easily identified through outliers, while additionally the margin of the breakdown values is visible by the width of the box, including the value for the median.

Furthermore, the breakdown field, defined as the breakdown voltage divided by the initial film thickness, rather than the breakdown voltage, is introduced, because it allows for a better comparison with published measurements conducted on samples with different membrane thicknesses. The small thickness change when the voltage is applied is not taken into account for the calculation of the electric field.

**Figure 4:**
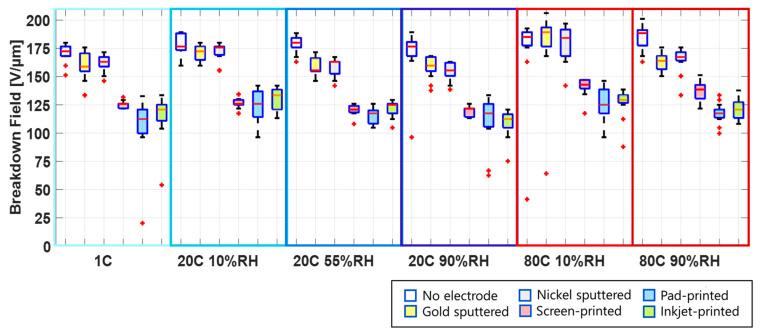
Breakdown field of Elastosil 2030/20 at different environmental conditions for pure silicone film (no electrode) and for film with electrodes applied using different manufacturing methods.

Figure 4 gives a comprehensive overview of the test results for each electrode type under all of the environmental conditions. The experiments were conducted at low, ambient, and high temperatures: 1 °C, 20 °C, and 80 °C. The ambient and high temperatures are tested each at low relative humidity (10% RH) and high relative humidity (90% RH). The difference in water content between the low and high humidity at 1 °C is only 4 g/m³, which is outside of the range adjustable in the climate chamber. Therefore, the results in Figure 4 for 1 °C are shown with an unspecified relative humidity.

Depending on the environmental conditions and the manufacturing method of the electrodes, breakdown fields ranging from 100 V/µm to 200 V/µm are measured. It is important to point out that the samples are pre-stretched bi-axially by 30%, and therefore the breakdown field is higher than what is expected for un-stretched samples. The influence of pre-stretching on the breakdown field is reported in different studies [25,43,44,45].

From Figure 4, one sees that the breakdown field for films without electrodes as well as films with sputtered electrodes is significantly higher than for samples with carbon black electrodes, for all environmental conditions.

### 3.1. Influence of Temperature and Humidity on Breakdown Behavior

**Figure 5:**
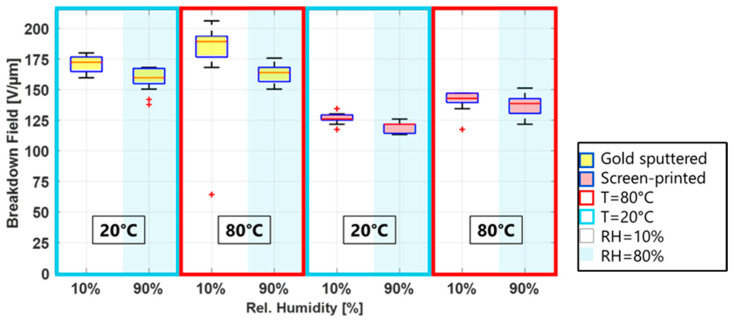
Influence of temperature (red and blue box) and relative humidity (light blue background) on the breakdown field of silicone film Elastosil 2030/20 for gold electrodes and screen-printed carbon black electrodes.

Even though breakdown fields vary between electrode materials and deposition methods, two trends are apparent for all electrode types: the breakdown field increases with an increasing temperature and decreases with increasing humidity. This is shown in Figure 5, where two temperatures at low and high humidity are displayed for a metal and a carbon black electrode. These two observations will be discussed in the following sections.

#### 3.1.1. Increase of Breakdown Field with Increasing Temperature

The exact composition of Elastosil 2030 is not known to the authors, but it is assumed that it mainly consists of polydimethlylsiloxan (PDMS). PDMS is a silicone polymer with a long backbone of alternating units of silicon and oxygen (Si-O) and two side chains of methyl on each silicon. The silicone molecule is helical, and intermolecular forces are low, hence easy rotational movements along the backbone are possible. The tetrahedral structure of the silicon in the chains prevents tight packing, thus the free volume in PDMS is high. Additionally, the methyl groups can rotate freely around the backbone [46,47], allowing for even more flexibility. How this composition is affected by temperature change can be explained using the free energy *F* of the system, defined in Equation (1), with *U*: Internal Energy, *T*: temperature, and *S*: the entropy [48].

(1) F=U−TS

The most stable condition is when the free energy is minimized. Silicone elastomers behave nearly entropy elastic above the glass transition temperature (−126 °C for Elastosil 2030), and therefore the contribution of the internal energy to the free energy can be neglected in our tested temperature range. From Equation (1), an increase in temperature and entropy will be more energetically favorable. An increasing temperature causes the PDMS molecules to move and rotate more freely and increase in entropy. This state has two consequences for the breakdown behavior. One is a stiffening effect and hence increase in Young’s modulus [49]. The other one is a prolonged breakdown through the membrane due to the high activity of the chains, which was observed by Du et al. in their investigation about the treeing characteristics at different temperatures [50].

A different influence of the temperature on the breakdown behavior for different electrode materials was not observed.

#### 3.1.2. Decrease of Breakdown Field with Increasing Relative Humidity

With increasing humidity at a constant temperature, a reduction in the breakdown field was observed for all electrodes. The high water vapor permeability of 3000 g/m²/24 h for Elastosil 2030/20 μm, [51], a result of the high free volume, allows for the water vapor to rapidly diffuse into and through the membrane. The free volume is a function of the temperature, and when the temperature increases, the free volume and the permeability increase as well [52]. Furthermore, the polymer chains become more mobile, and the diffusion of the water molecules is favored. The mobility of the water vapor molecules significantly depends on the temperature as well. Thus, higher temperatures mean higher gas mobility which subsequently results in a higher gas diffusivity and permeability [53].

Depending on the temperature and humidity, the membrane can absorb 0.1 to 0.25 wt.% of water [54,55]. The absorbed water molecules in the membrane lower the dielectric properties of the material because the water introduces additional charge carriers and thus breakdown takes place at lower breakdown fields.

Figure 5 shows the breakdown fields for metal (gold) electrodes and carbon black (screen-printed) electrodes for two temperatures, at low and high humidity. The lower breakdown field for higher humidities is clearly visible for both electrodes. However, while the median difference (calculated by subtracting the medial breakdown at 90% RH from 10% RH and divided by the 10% RH value times 100) for the CB electrodes is around 3% for both temperatures, it is significantly higher, 13.5%, for the gold electrodes at 80 °C and 90% RH.

### 3.2. Effect of Electrode Manufacturing-Induced Mechanical Actions on Breakdown Behavior

**Figure 6:**
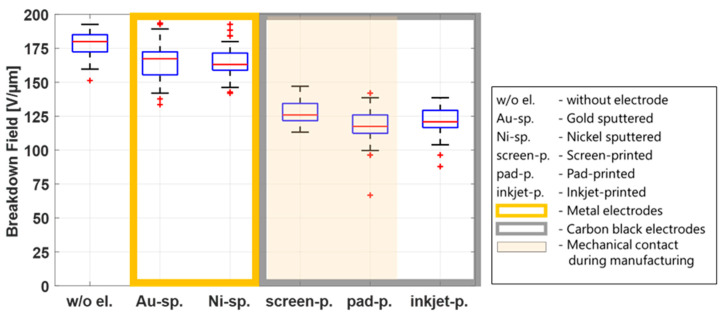
Summarized breakdown field results from all temperature and humidity tests conducted in Figure 4 subdivided into samples without electrodes, metal electrodes, and CB electrodes.

Section 3.1 illustrated the influence of the environmental conditions on the breakdown field and reported how the breakdown field is different for different electrode types for a given environmental condition. This difference can either be due to processes during manufacturing or due to specific electrode properties and their influence on the film. This section explores whether the manufacturing process is responsible for a mechanical change of the film. Figure 6 shows a breakdown plot for all electrode types, combining data from all temperatures and humidities in one box for each electrode type (90 data points per electrode).

The sputtering process and the inkjet-printing process are non-contact manufacturing methods. During screen printing and pad printing, direct mechanical contact with the film is necessary. In the screen-printing process, the mesh touches the film, and a squeegee applies the electrode material through the mesh. In the pad-printing process, the pad, which transfers the electrode material onto the film, stamps the electrode material directly onto the film. Figure 6 shows higher breakdown fields for the sputtered electrodes, but the results for the inkjet-printing electrodes are in the same order of magnitude as for the contact processes screen printing and inkjet printing, indicating that the mechanical impact is not a decisive parameter that influences the breakdown behavior. Because a breakdown field reduction is observed whenever electrodes are applied—compared to the reference samples without electrodes—other parameters responsible for this phenomenon are discussed in the following sections.

### 3.3. Influence of Electrode Stiffness

Figure 6 shows that the breakdown field of the samples with metal electrodes (gold and nickel) is significantly higher than that of the samples with CB electrodes, regardless of temperature or humidity. Therefore, the following investigations of possible parameters influencing the breakdown behavior of the film will only be discussed under standard environmental conditions (20 °C and 55% RH).

An important difference between metal and CB electrodes is the stiffness of the material. When a voltage is applied, the Maxwell stress induces a thinning of the membrane and subsequently an increase in the electric field. In an ideal silicone film, where incompressibility is assumed, the thinning of the membrane will result in an area extension of the film.

**Figure 7:**
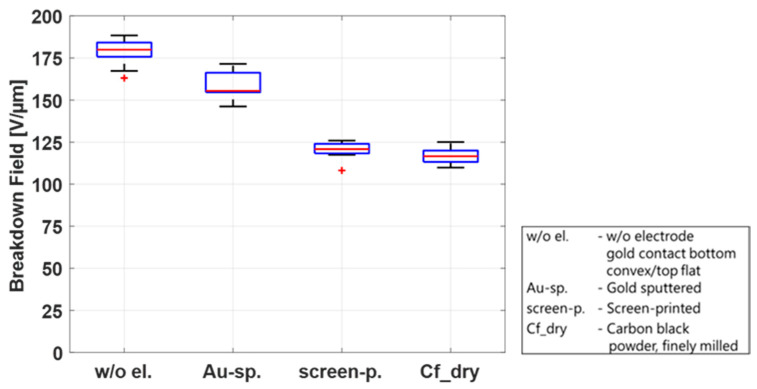
Comparison of electrodes with different stiffnesses (external-measurement electrodes, sputtered gold electrodes, screen-printed CB/PDMS electrodes, and finely milled CB powder) and their influence on the breakdown field at 20 °C/55% RH.

Two parameters generate an electric field increase: the thinning of the dielectric membrane due to the Maxwell stress and the voltage increase due to the experimental procedure. While soft CB electrodes allow for both of these mechanisms to occur, gold electrodes are extremely stiff and hence impede the area extension. Because of the membrane’s incompressibility, this subsequently strongly suppresses the thinning of the material under the application of a voltage. In this way, the electric field in the gold electrode case only increases due to the voltage increase and is thus lower than the electric field, resulting from the same voltage with soft CB electrodes. To confirm this theory, two limiting cases are investigated. One case is a very soft electrode, consisting only of finely milled CB powder. The other is a rigid electrode. Here, the external measurement electrodes are used, which adhere to the pure film when voltage is applied, thus restricting expansion. For this case, the highest breakdown field is expected. The CB powder adds nearly no stiffness to the film; thus, the lowest breakdown field is expected. Figure 7 shows the breakdown field for the four discussed configurations. The results confirm the theory that with an increasing rigidity of the electrode, the breakdown field will also increase.

Another possible mechanism for the higher breakdown voltage in the case with only measurement electrodes might be the fact that the contact surface is reduced in comparison to sputtered electrodes due to surface roughness and potential micro-sized air bubbles.

The previous sections explained the factors that are mainly responsible for the different breakdown behavior of metal and carbon black electrodes. The following sections will focus on the material parameters of carbon black electrodes and their possible influence on the breakdown behavior. Metal electrodes are not included in these sections.

### 3.4. Carbon Black Electrodes: Influence of Solvents with and without Carbon Black

During the manufacturing process of carbon black electrodes via screen printing, pad printing, and inkjet printing, solvents are necessary to provide the electrode material with the viscosity required for the respective process. Even though the solvents are fully evaporated when the electrodes are cured, an influence on the breakdown during the manufacturing is possible. This section will investigate a possible influence of solvents on the breakdown behavior of the DE membrane under standard environmental conditions.

**Figure 8:**
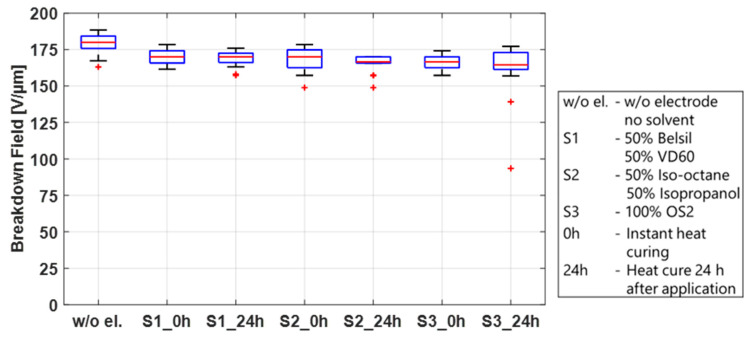
Influence of different solvents and their dwell time before heat curing at 150 °C on the breakdown field of Elastosil 2030/20 µm.

First, the breakdown effect of the three solvent mixtures used for screen printing, S1 (50% Belsil/50% VD60), pad printing, S2 (50% Iso-octane/50% Isopropanol), and inkjet printing, S3 (OS2), was studied. These tests were conducted on twelve test frames, four for each mixture, pre-stretched with Elastosil 2030/20 µm, identical to the frames prepared in the sections above. A total of 0.2 mL of each solvent mixture per test frame was applied by a syringe along the length of the frame and immediately distributed over an area of 110 mm × 20 mm using a spatula. Immediately after application, mixtures S1 and S3 led to significant swelling of the membrane, whereas less swelling but the formation of micro bubbles on the surface of the film was observed using mixture S2. To determine if these phenomena influence the breakdown behavior and also if they are dwell-time dependent, two samples of each mixture were heat cured immediately and two samples were first stored for 24 h before heat curing at 150 °C for one hour. The samples were then tested in the breakdown tester, and the results are shown in Figure 8. For comparison, the results of untreated samples are also included in this figure. No significant differences in the breakdown fields are observed between solvent mixtures, regardless of whether the solvents remained on the sample before curing or were heat cured immediately. Compared to the breakdown field of the untreated samples, only a slightly lower breakdown field (~10 V/µm between medians) is visible. This indicates that the swelling of the membrane after application and heat curing is only temporary and has no major impact on the membrane. The solvent by itself is therefore not the sole parameter responsible for the different breakdown behavior of sputtered metal electrodes and carbon black electrodes, but it may have an influence when carbon black is added to the mixture.

**Figure 9:**
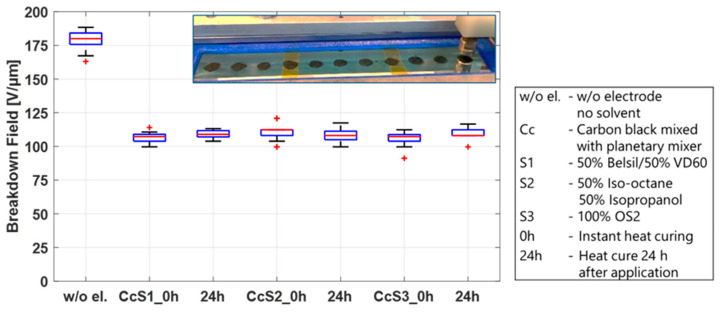
Influence of different solvents mixed with CB powder (without milling), dwelling time before heat curing on the breakdown behavior, and a picture with electrodes applied by hand using a stencil.

To investigate the influence of the solvent mixtures in the presence of carbon black, 0.5 g of CB was added to 6 g of each solvent mixture and processed in a planetary mixer. A planetary mixer was chosen because the different volatilities of the solvents would result in different evaporation rates when processed in a three-roll mill. The CB/solvent mixtures were manually applied using a stencil and a spatula. As in the test conducted above, two frames of each mixture were immediately heat cured, and two frames were stored 24 h before heat curing at 150 °C for 1 h. A picture of the electrode dots on the film as well as the breakdown results are shown in Figure 9. The addition of CB to the solvents has a significant impact on the breakdown field, regardless of the solvent mixture or dwell time, lowering the breakdown field by 40%. This phenomenon can be explained by two different mechanisms. On the one hand, the soft electrode layer covers the entire surface and thus detects all irregularities and changes in the thickness of the film, which are specified by the data sheet to +/−5%. The breakdown field will therefore always be determined by the thinnest spot of the film. Second, as discussed above, when solvents are applied to the membrane, swelling of the membrane or the forming of small bubbles on the surface are observed. When CB particles are present, it allows the particles to embed into the membrane, effectively thinning the dielectric layer. A calculation of the thickness reduction of the dielectric layer based on the difference in the breakdown field of the pure film (median 180 V/µm) and the film with the CB/solvent electrodes (median 110 V/µm) results in a reduction of 4.5 µm, or a 2.25 µm layer of embedded CB particles on each side. Even though the primary aggregate size of conductive carbon blacks is in the nanometer range, conglomerates > 150 nm up to micrometer structures form when solvents are added [56,57].

**Figure 10:**
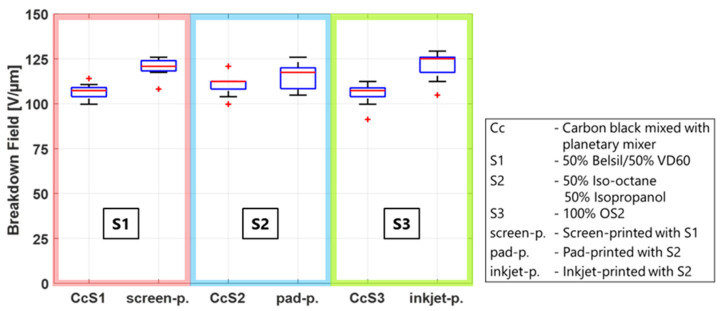
Influence of three solvent mixtures in CB powder and processed in their respective manufacturing method on the breakdown behavior.

The results of Figure 9 are now compared to the results at the standard environmental conditions of Figure 4 to determine whether the lower breakdown field of the manufactured CB electrodes is solely a function of the solvent/CB mixtures, or if additional material parameters influence the breakdown behavior as well. Therefore, each solvent mixture is compared to the respective manufacturing method using this solvent. The results are shown in Figure 10, where the CB/solvent and manufacturing method for each solvent are combined in one box for better comparability. It is clearly visible that the breakdown field of all three manufactured electrodes is considerably higher than that of the CB/solvent mixture, indicating that not only do carbon black and solvents have an impact on the breakdown behavior but also that other manufacturing or material parameters are relevant as well. Two additional parameters possibly influencing the breakdown are (i) the fineness of the carbon black particles used in the process and (ii) the amount of PDMS added to the electrode material. Both will be investigated in the next sections.

### 3.5. Influence of Carbon Black Processing

Section 3.4 discussed the importance of investigating a possible impact of the fineness of the CB particles in the applied electrodes on the breakdown behavior. Because CB particles agglomerate when blended with most liquids, a grinding process is necessary to break down the agglomerates to smaller sizes. The grinding process was different in the three manufacturing methods. Though the process is explained in Section 2.1 and in the references, a short comparison is necessary for a better understanding: the CB/solvent electrodes used in Section 3.4 were mixed using a planetary mixer; the CB for pad printing was also mixed using a planetary mixer, but steel balls were added to increase grinding. The CB in the screen-printing electrodes was first mixed in a planetary mixer and subsequently milled in a three-roll mill. Lastly, the CB for the inkjet formulation was first ground in a three-roll mill and subsequently sonicated in an ultrasound bath, with a 10 min waiting period for the larger particles to settle down before decanting. The fineness of the CB mixtures increases from CB/solvent to inkjet formulation in the order above.

The experiments were conducted on electrodes consisting of carbon black, and the solvent with the lowest vapor pressure, S1, was processed with the planetary mixer as an example of a coarser blend and a three-roll mill as an example for a finer blend. To investigate the influence on the membrane when no swelling from the solvents occurs, the experiments were additionally conducted with a CB/distilled water mixture. Both sample sets were prepared in the same way and heat cured at 150 °C for 1 h.

**Figure 11:**
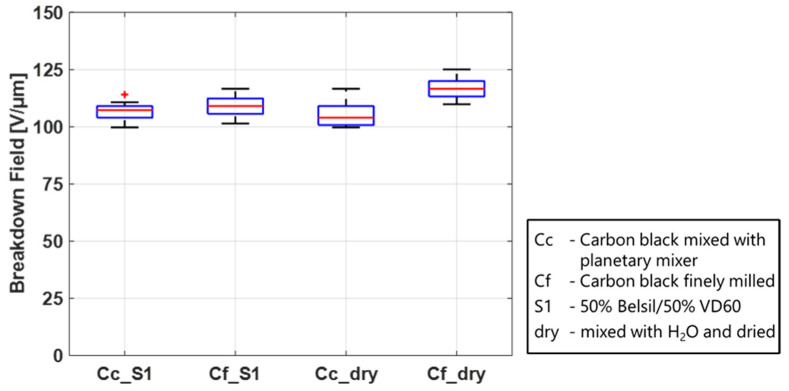
Influence of carbon black particle fineness on the breakdown behavior of Elastosil 2030/20. Particles were either mixed in a three-roll mill for a very fine homogeneous distribution or mixed using a planetary mixer, obtaining a slightly coarser structure.

The results in Figure 11 show a higher breakdown field when the carbon black is more finely milled than in the coarser samples. While this effect is only small in the electrodes with solvents, it is considerably more pronounced in the electrodes without solvents. A possible explanation could be that due to the swelling of the membrane, when in contact with solvents, CB particles can be embedded in the surface layer of the PDMS membrane, and the difference between very fine and coarser particles will not be as pronounced. Without solvents and no swelling, however, the particles will stay on the surface of the membrane. Larger particles could damage the film more when an electric field is applied, probably due to the sharp edges and higher imprint. If the CB powder is very fine, the damaging effect on the film is likely reduced and a higher breakdown field than for the fine CB particles and solvents is achieved. The presence as well as the processing of the carbon black is an important parameter to influence the breakdown behavior of a DE.

### 3.6. Influence of Silicone Content (PDMS)

The influence of solvent and carbon black has been investigated, and the last important material parameter to study is the silicone content. The silicone content in the electrode mixtures varies depending on the manufacturing method. The highest PDMS content—90 wt.% after curing—is used in pad printing. The screen-printed electrodes consist of 75 wt.% PDMS, and the inkjet electrode material is not mixed with PDMS at all but with a silicone polyglucoside dispersant (~60 wt.% after solvent removal).

**Figure 12:**
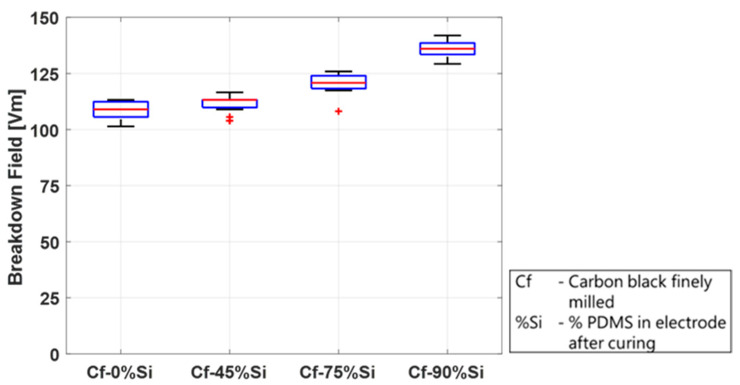
Influence of silicone content in the electrode matrix on the breakdown behavior of Elastosil 2030/20 at 20 °C/55% rel. humidity in wt.%. All electrodes except Cf-0%Si are applied by the screen-printing method.

The study in this section was conducted on mixtures containing four different PDMS concentrations—0 wt.%, 45 wt.%, 75 wt.%, and 90 wt.%—after curing (without solvents). A stock mixture of CB and PDMS was prepared using a planetary mixer and was subsequently milled in a three-roll mill. The required test concentrations were then blended with PDMS using the planetary mixer. The electrode dots containing PDMS were applied using the screen-printing method. The electrodes without silicone were not processable in a screen printer and were applied as described above using a stencil. All the samples were cured at 150 °C for one hour. The results are depicted in Figure 12 and show a significant impact of the PDMS content in the electrodes on the breakdown behavior.

The breakdown field increases with an increasing PDMS content, from a median of 109 V/µm without silicone to 136 V/µm in the mixture with 90 wt.% silicone. This could be explained by the fact that the higher the silicone content, the more the particles are embedded in the silicone structure, and damage due to sharp particles is reduced. Furthermore, less CB particles are directly located on the surface but are enclosed in the dielectric matrix. This proposed explanation is in agreement with the results of Section 3.5, where finely milled CB dust present on the top of the film and not embedded in the silicone also leads to lower breakdown fields. It should be mentioned, however, that this is only an observation regarding the breakdown field. A higher PDMS/carbon black ratio has not only the advantage of the higher breakdown field and better attachment to the film but also the disadvantage of a lower electrical resistance, as discussed in detail by Willian et al. [58].

## 4. Conclusions

This paper presented results on the dielectric breakdown of silicone-based electroactive polymer actuators to give a comprehensive understanding about breakdown-influencing parameters. A particular focus was on the effects of electrode composition and electrode manufacturing. These results will allow for a comparison and interpretation of results from different published studies, using these results to better design given applications. As previous work [19] indicated a significant impact of the presence of electrodes on the PDMS membrane, it is important to understand which parameters influence the electric breakdown behavior. This paper focused on a systematic study of different electrodes including gold- and nickel-sputtered electrodes as well as carbon black-based electrodes applied by widely used manufacturing methods such as screen printing, pad printing, or inkjet printing. In addition to a systematic comparison of the different materials, the present work also studied the effect of environmental parameters, such as temperature and humidity, on the breakdown behavior.

We found that adding electrodes lowers the breakdown field compared to films without electrodes. The effect, however, is low for sputtered metal electrodes but significant when carbon black electrodes are applied, reducing the breakdown field by up to 30%. Possible parameters responsible are identified as the mechanical impact during manufacturing, the stiffness of the electrode, and material parameters. Material parameters include the used solvent, carbon black processing, and the silicone content. They were investigated and discussed in detail.

The mechanical impact due to direct contact with the membrane during screen printing and pad printing has no effect on the breakdown behavior compared to inkjet printing, where no direct contact is present. The influence of the electrode stiffness is clearly visible, showing that breakdown tests using stiff metal electrodes yielded a higher breakdown field.

Further investigations should focus on material parameters used for carbon black electrode manufacturing, because these electrodes are common for actuator and sensor applications and are applied by fast and scalable processes. Different manufacturing methods require different solvents, thus the solvent mixtures used during the three manufacturing methods were investigated separately. The data indicate only a minor influence of the solvents on the breakdown field, regardless of the dwelling time on the film before curing. When carbon black is added to the solvents, however, the breakdown field is reduced by up to 40%. This effect is slightly improved when the fineness of the carbon black particles is increased, e.g., through processing in a three-roll mill. Another important parameter influencing the breakdown behavior is the silicone content in the electrode material. The breakdown field is significantly increased when silicone is added to the electrode material and increases with increasing silicone content.

The study on the influence of various environmental conditions was carried out for all types of electrodes with temperatures between 1 °C and 80 °C and humidities ranging from 10%RH to 90%RH. An increasing breakdown behavior with an increasing temperature and decreasing humidity was observed for all electrode types.

## Figures and Tables

**Table 1:** Application range of electrode manufacturing methods for DEAs used in this study and associated advantages/disadvantages.

	Advantage	Disadvantage
Screen-printing	Very fast process, ready up-scaling to mass production Printing of small and large areas High acceptable range of ink viscosity (500–10,000 mPas)	New screen for each design → not ideal for prototyping Material waste for prototyping due to large minimum amount of ink needed for first print Can only print on flat or rounded surfaces Mechanical impact of screen on DE film
Pad-printing	Printing on irregular shaped surfaces possible Fast process Medium range of acceptable ink viscosity (1500–2000 mPas)	Stencil necessary → not ideal for prototyping Cannot print on large areas Mechanical impact of soft pad on DE film
Inkjet-printing	No screen or stencil necessary Ideal for prototyping Contactless process—no mechanical impact	Very low range of acceptable ink viscosity (10–20 mPas) Ink needs high solvent content Clogging of nozzle requires frequent cleaning procedures Slow process, poorly suited for mass production
Sputtering	Nanometer-thick high-conductivity electrodes Microscale actuator designs possible with subsequent laser ablation Ideal for micro-structures incl. connections using laser ablation	Slow and complex process for laboratory sputtering systems, involving a vacuum step Pre-stretch of film required for sputtering and subsequently releasing to avoid cracks → DE operation preferrable within pre-stretched range High investment cost for mass production

**Table 2:** Temperatures and relative humidities of test conditions.

Temperature and % RH		
1 °C—undefined % RH	20 °C 10% RH	80 °C 10% RH
	20 °C 55% RH—defined as standard environmental condition	
	20 °C 90% RH	80 °C 90% RH

## Data Availability

The data presented in this study are available on request from the corresponding author.
